# Study of Sedative-Hypnotic Effects of *Aloe vera *L. Aqueous Extract through Behavioral Evaluations and EEG Recording in Rats

**Published:** 2016

**Authors:** Fatemeh Abdollahnejad, Mahmoud Mosaddegh, Sanaz Nasoohi, Javad Mirnajafi-Zadeh, Mohammad Kamalinejad, Mehrdad Faizi

**Affiliations:** a *School of Traditional Medicine, Shahid Beheshti University of Medical Sciences, Tehran, Iran.*; b *Department of Pharmacology and Toxicology, School of Pharmacy, Shahid Beheshti University of Medical Sciences, Tehran, Iran. *; c *Deptartment of Physiology, Faculty of Medical Sciences, Tarbiat Modares University, Tehran, Iran. *; d *Department of Pharmacognosy, School of Pharmacy, Shahid Beheshti University of Medical Sciences, Tehran, Iran.*

**Keywords:** *Aloe vera*, Insomnia, Sedative-Hypnotic effects, Electroencephalography, Electromyography

## Abstract

In this study, we investigated the sedative and hypnotic effects of the aqueous extract of *Aloe vera* on rats. In order to evaluate the overall hypnotic effects of the *Aloe ver*a extract, open field and loss of righting reflex tests were primarily used. The sedative and hypnotic effects of the extract were then confirmed by detection of remarkable raise in the total sleeping time through analysis of electroencephalographic (EEG) recordings of animals. Analysis of the EEG recordings showed that there is concomitant change in Rapid Eye Movement (REM) and None Rapid Eye Movement (NREM) sleep in parallel with the prolonged total sleeping time. Results of the current research show that the extract has sedative-hypnotic effects on both functional and electrical activities of the brain.

## Introduction

Insomnia is a sleep disorder characterized by inability of efficiently falling into and staying asleep to restore normal states of energy and wakefulness (1, 2). Reportedly one-third of the general adult population (2) particularly females (3) experience insomnia at some point in their lives. Although the prevalence of insomnia estimates very largely based on the diagnostic methods (4), there is no doubt on the enormous economic impact of sleep disorders (5, 6). Insomnia may be associated with obesity (7), increased risk for metabolic syndrome (8), coronary artery disease (9, 10), depression (11), and anxiety (12, 13), as well as being a cause of concentration and memory problems (14, 15).

Given primary insomnia seems to be the most common diagnosis (2), intensive pharmacological treatment is inevitable in many patients. Although efficient therapeutics like benzodiazepines are available for insomnia, clinical applications are limited because of concerns about their potential abuse, dependence, and adverse effects (16). Behavioral therapies also have empirical evidence for relieving insomnia, however they have remained generally unemployed because of the time-intensive nature and need for expert trainees for effective application (17). Regarding the limitations and unfeasibility of existing therapies of insomnia, alternative and traditional medicine can be interesting as new treatment of insomnia.

Old materia medicas offer a variety of remedies for sleeping ailments, namely *Crocus sativus* (safron), *Egyptian lotus*, *Solanum nigrum* and *Aloe vera* (*A. vera*) (18). *Aloe vera* L. (*Aloe barbadensis* Miller) named as Sabr-e-zard (19), a succulent plant belonging to the Liliaceae family is amongst well known Iranian traditional medicine(20).

*A. vera* is one of the most widely used herbal medicines well known because of its local anti-inflammatory and healing properties. Besides being one of the most popular herbal medicines worldwide (21), it is also increasingly used in food industries (22). The clear gel isolated from the plant leaves, which has a variety of nutrients and bioactive molecules, is widely used in skin care, cosmetics and as “nutraceuticals” (23). Systemic consumption has also been empirically confirmed to improve a variety of health elements including immune system (24), angiogenesis (25), and gastrointestinal integrity (26). 

*A. vera* therapeutic efficacies are relied on various bioactive compounds. Amylase and salicylates for instance render the extract as an anti-inflammatory and antibacterial agent (27). Sedative and hypnotic effects of *A. vera* have been reported in several Iranian and international old pharmacopoeias (18, 20, 28). *A. vera* extract contains certain biochemical components such as flavonoids (29) and amino acids (30) which have been previously documented to affect sleep quality.

Regarding many surveys implying beneficial effects of *A. vera* in CNS diseases (31, 32, 33), the present work aims to find experimental support for the reported hypnotic effects of *A. vera* in traditional medicine. In order to do that, the effect of aqueous extract of *A. vera* leaves on locomotion and pentobarbital induced sleeping of rats was investigated. More details about influence of *A. vera* on the sleep architecture were obtained through investigation of Electroencephalogram (EEG) and Electromyogram (EMG) recordings of the animal. 

## Results and Discussions

*Behavioral examination of sedative-hypnotic properties of A. vera aqueous extract*

*Investigation of pentobarbital-induced loss of righting reflex*

**Figure 1:**
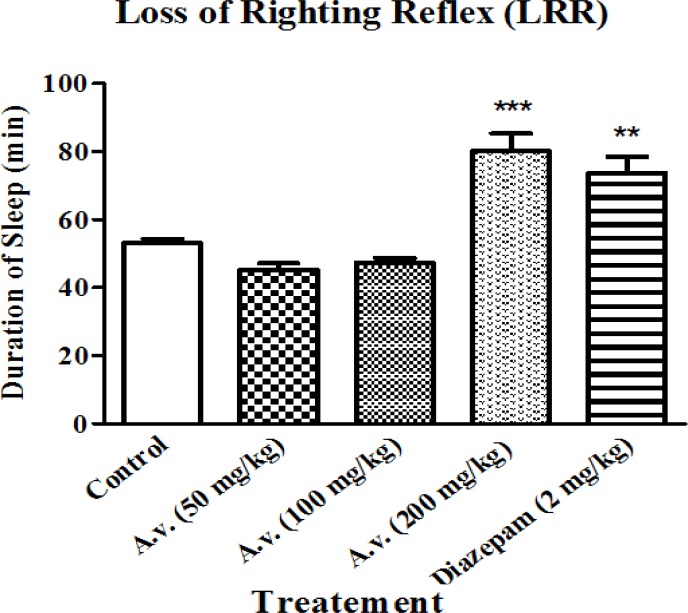
Aloe vera (*A. vera*) aqueous extract prolonged pentobarbital induced loss of righting reflex. Rats received pentobarbital (40 mg/kg, i.p.) 30 min following Aloe vera extract (50, 100, 200 mg/kg, i.p.) or diazepam (2 mg/kg, i.p.). *A. vera* hypnotic effects were evaluated based on increasing the sleeping time in test animals. Data are represented as mean ± SD (n=6). *** p < 0.001, ** p < 0.01 compared to control group

As the present work essentially aims to investigate hypnosis in response to *A. vera* administration, changes in loss of righting reflex was considered to estimate hypnotic effects of the extract. Administration of the extract did not influence onset of pentobarbital induced sleeping (data are not shown here) but prolonged the representative loss of righting reflex as the main characteristic of hypnotic agents. As it is presented in Figure 1, administration of the extract (200 mg/kg) led to prolonged loss of righting reflex compared to the control group of animals. Prolongation of loss of righting reflex in the animals was statistically equal to that of the animals which had received diazepam (2 mg/kg) as the positive control. 

*Investigation of locomotion activity*

**Figure 2:**
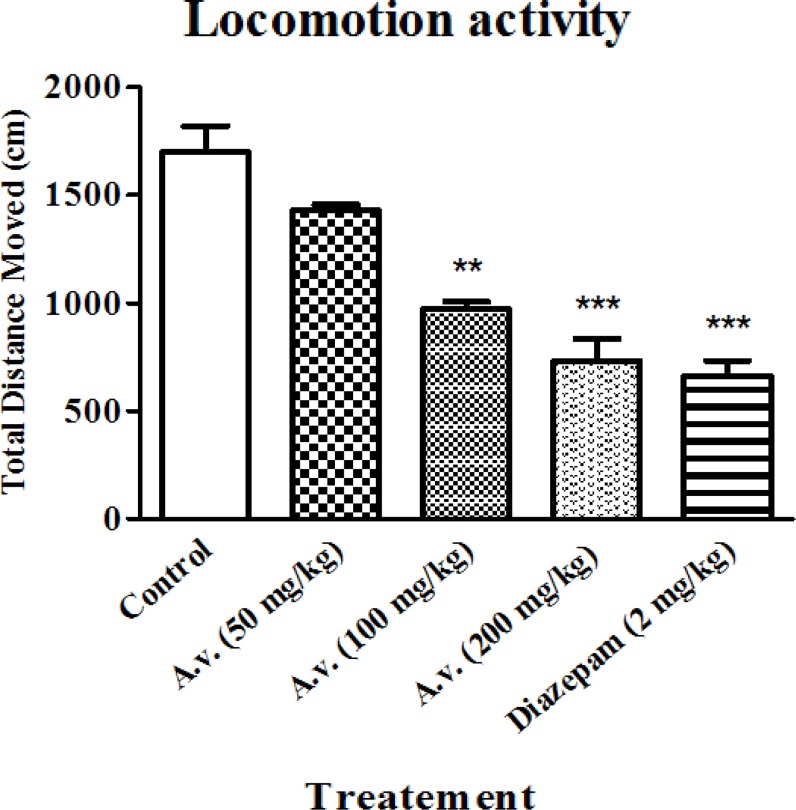
Aloe vera (*A. vera*) aqueous extract repressed animals' locomotor activity. 30 min after Aloe vera extract (50, 100, 200 mg/kg, i.p.) or diazepam (2 mg/kg, i.p.), animals were subjected to open field test and animals’ total distance moved were compared as an indicative for locomotor activity. Data are represented as mean ± SD (n=6). *** p < 0.001, ** p < 0.01 compared to control group

While hypnotic properties of the extract were primarily determined in loss of righting reflex examination, locomotion activity alteration was also considered as an index for the sedative effect. The open field results in conjunction with data obtained from loss of righting reflex test, confirmed sedative-hypnotic effects of the extract. Figure 2 shows significantly repressed locomotion activity of the animals after administration of the extract (100 and 200 mg/kg) probably as a result of its sedative effect. The results show that administration of the extract at dose of 200 mg/kg is as efficient as diazepam (2 mg/kg) in suppressing the locomotor activity of the animals.

*Investigation of sleep parameters*

**Figure 3:**
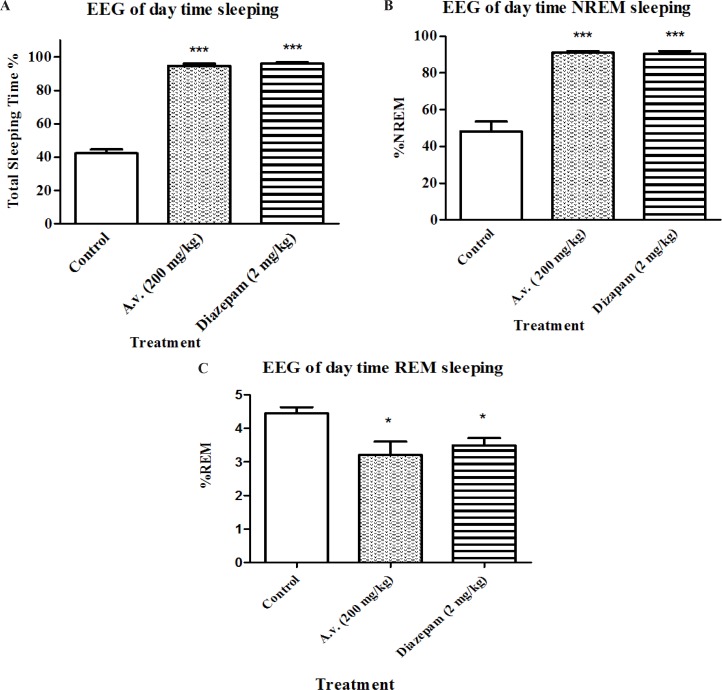
Impact of Aloe vera (*A. vera*) extract administration on electroencephalographic architecture of sleep. Day-time EEG and EMG recordings were conducted in freely moving rats following 30 min post Aloe vera or diazepam administration for 4 hours. Accordingly, Aloe vera aqueous extract (200 mg/kg, i.p.) prolonged total sleeping time (A) as well as NREM sleep (B) while reduced sleeping time spent in REM (C). Data are represented as mean ± SD (n=6). * p < 0.05, ***p < 0.001 compared to control group

In order to be able to define significant sedative-hypnotic effects for the extract, results of loss of righting reflex test, as a widely used screening test for hypnotic compounds, needs to be confirmed by complementary methods. Therefore EEG recording was performed to determine duration of awaking, NREM and REM sleep states. Figures 3 A, 3 B and 3 C represent changes in sleep pattern of the animals which received the *A. vera* extract (200 mg/kg) in four hours during daytime. Animals treated with *A. vera* had following sleep parameters: total sleep time [F(2,15)=358, p < 0.05], percent of REM sleep [F(2,15)=15.5, p < 0.05] and percent of NREM sleep [F(2,15)=14, p < 0.05]. Results of Bonferroni’s post-test of EEG recording show that A.vera as well as diazepam increased the sleeping time (p < 0.001) and NREM sleep duration (p < 0.001) and decreased REM sleep (p < 0.001) compared to the control group.

Active pharmacological ingredients of *A. vera* , concentrated in the gel and rind of the plant leaves have been evidently shown to exert analgesic, anti-inflammatory, antioxidant and anticancer effects (38). Amongst identified therapeutic indications for *A. vera* in folk medicine, treatment of insomnia has not been addressed yet by any scientific experiment. The present work evaluates sedative and hypnotic effects of *A. vera* through performing open field as well as pentobarbital induced-sleeping prolongation, as screening tests, on rats. Whereas open field test is not specific for sedation–related behaviors, suppressed locomotion activity in conjunction with hypnosis determined by prolongation of loss of righting reflex in animals, may highlight sedative properties of the *A. vera* extract. Increase of total sleeping time observed in investigations of EEG recordings of the same animals during the tests, could provide rational proof for the suppressed locomotion reported in our behavioral examinations. 

To our knowledge, the only relevant survey by now has been a multicentre clinical open study on a topical moisturizer preparation containing *A. vera* extract for which 100% sleep improvement have been reported in subjects bearing atopic dermatitis (39). According to the study design however, this could be simply accounted for the relief from itching discomfort probably resulting from histamine release suppression by *A. vera* (26, 38) .

The essential characteristic of sleep is full reversal with efficient external stimuli. Therefore, virtual hypnotic impact of bioactive compounds could be accurately evaluated by EEG recording in normal, rather than drug induced sleeping animals. The EEG records of our day-time sleeping studies illustrate substantial electrophysiological effects of *A. vera* on sleep length that seemingly were efficient enough to produce the behavioral responses we observed in open field and loss of righting reflex test.

Besides prolongation of total sleeping time, *A. vera* administration led to a significant shift toward more NREM sleep. Such alterations in REM and NREM sleep parameters may provide useful data about outcomes of certain therapeutics. REM and NREM sleep are two distinct stages which are different not only in cerebral electrophysiological status but also in simultaneous resting muscular tonus. That is whilst thought-like mental activity takes place during NREM sleep. REM sleep is mostly associated with hallucinations concurrent with muscular paralysis. Several investigations on memory performance have also revealed that REM sleep contributes to consolidation of procedural memory (40) while NREM improves declarative memory (41).

The hypnotic activity of herbal medicines has been frequently attributed to different phytochemicals compounds such as flavonoids and saponines (29, 42). In the case of protein rich plants however, presence of certain amino acids may be of prominent importance (43, 44). 

Versatile non-amino acid neurotransmitters such as acetylcholine and catecholamine are involved in governing normal sleep quality. That is centrally acting anticholinergic, dopaminergic, noradrenergic, and serotonergic agents cause a decrease in duration and density of REM sleep (45, 46). Recent evidence has elucidated significant changes in cerebral neurotransmitters in mice treated with *A. vera* extract of which diminished levels of nore-epinephrine and serotonin are conspicuous (47). Regardless of probable impact on sleep parameters, we postulated such alterations might not apply to our set of experiments in which no long-term dosing protocols were included. 

*A. vera* may be expected to elevate acetylcholine levels based on some reports implying its choline-esterase inhibition property (48). It has been shown that REM sleep duration is decreased by central cholinergic system augmentation and parasympathetic tone is dominant in NREM sleep (49, 50). The observed changes in REM and NREM sleep can be partly explained by presence of compounds with anti-choline-esterase activity in *A. vera*. The implication of any of the mentioned neurotransmitters however, needs further elucidation as our experiments did not include any contributing examination. 

## Conclusion

Several investigations have provided experimental evidences for CNS-ailments that have been traditionally claimed to be improved by *A. vera* administration of which convulsion (51), cerebral ischemia (25) and multiple sclerosis (33) seemingly are the foremost ones. Present work provides positive evidences which support sedative and hypnotic effects of *A. vera* extract obtained by corresponding traditional method described in folk medicine. While investigating the corresponding properties of cold-dried extracts seem extremely intriguing for further works, our results also remained a question whether *A. vera* exerts ameliorating effects in the context of insomnia in human.
